# Manganese Uptake by A549 Cells is Mediated by Both ZIP8 and ZIP14

**DOI:** 10.3390/nu11071473

**Published:** 2019-06-28

**Authors:** Ivo F. Scheiber, Neftali Ortega Alarcon, Ningning Zhao

**Affiliations:** Department of Nutritional Sciences, The University of Arizona, Tucson, AZ 85721, USA

**Keywords:** manganese, ZIP8, ZIP14, lung, alveolar epithelia, transporter, inhalation exposure

## Abstract

The alveolar epithelia of the lungs require manganese (Mn) as an essential nutrient, but also provide an entry route for airborne Mn that can cause neurotoxicity. Transporters involved in Mn uptake by alveolar epithelial cells are unknown. Recently, two members of the Zrt- and Irt-like protein (ZIP) family of metal transporters, ZIP8 and ZIP14, have been identified as crucial Mn importers in vivo. ZIP8 is by far most abundantly expressed in the lungs, whereas ZIP14 expression in the lungs is low compared to other tissues. We hypothesized that Mn uptake by alveolar epithelial cells is primarily mediated by ZIP8. To test our hypothesis, we used A549 cells, a type II alveolar cell line. Mirroring the in vivo situation, A549 cells expressed higher levels of ZIP8 than cell models for the liver, intestines, and kidney. Quantification of ZIP8 and ZIP14 revealed a strong enrichment of ZIP8 over ZIP14 in A549 cells. Using siRNA technology, we identified ZIP8 and ZIP14 as the major transporters mediating Mn uptake by A549 cells. To our surprise, knockdown of either ZIP8 or ZIP14 impaired Mn accumulation to a similar extent, which we traced back to similar amounts of ZIP8 and ZIP14 at the plasma membrane. Our study highlights the importance of both ZIP8 and ZIP14 in Mn metabolism of alveolar epithelial cells.

## 1. Introduction

Manganese (Mn) is an essential nutrient required for the normal function of several physiologic processes including protein glycosylation, defense against oxidative stress, and gluconeogenesis [1,2]. Yet, excess Mn is neurotoxic, and elevated accumulation in the brain may result in manganism, an extrapyramidal disorder whose symptoms resemble those of Parkinson’s disease [3,4].

Under normal conditions, oral exposure is the major source for Mn absorption [5]. Manganese entering the body via this route is tightly controlled by regulating intestinal Mn absorption and hepatobiliary Mn excretion [6,7,8,9]. Chronic Mn toxicity due to the gastrointestinal absorption in individuals with no genetic susceptibility is not a major public health concern [10]. On the contrary, individuals exposed to high levels of airborne Mn, such as welders or miners that are exposed to Mn-rich fumes and dusts, are at increased risk to develop manganism, suggesting that absorption via the lungs and the olfactory system are important routes of exposure to Mn [3,4]. Olfactory transport, however, does not contribute significantly to the delivery of Mn to the striatum, the major target site for Mn neurotoxicity [11,12], suggesting that Mn absorption from the pulmonary tract primarily accounts for the neurotoxicity caused by inhalation of airborne Mn.

Comprising 99% of the surface area of the lungs, the alveolar epithelium is the largest surface of the body exposed to the environment [13,14,15,16,17]. Beside gas exchange, the alveolar epithelium functions as a physical barrier between the lumen and the underlying submucosa, to defend the tissue from environmental stressors such as particles, microorganisms, and toxins [16,17]. It consists mostly of Type I and Type II alveolar cells that are present in roughly similar numbers, although Type I cells cover about 96–98% of its surface [14,15,16,17]. Despite covering only a small portion of the alveolar surface, Type II alveolar cells contribute substantially to active ion transport within the alveolus [14,15].

Being in contact with both the blood and the alveolus lining fluid, alveolar epithelial cells can take up endogenous Mn as well as airborne Mn. To date, the molecular mechanisms governing Mn acquisition by alveolar epithelial cells remain unknown. Transferrin (Tf)/transferrin receptor (TfR) and divalent metal transporter 1 (DMT1)-mediated Mn uptake have been suggested to be involved in the uptake of airborne Mn [18]. However, interactions between ^54^Mn and Tf in the lung fluid of rats intratracheally instilled with ^54^Mn has not been detected, and the transport of intratracheally instilled ^54^Mn into the blood was unimpaired in Belgrade rats that lack functional DMT1 [18].

Recently, two members of the ZIP-family of metal transporters, ZIP8 and ZIP14, have been identified as crucial players in Mn metabolism in vivo [19,20,21]. Both transporters have previously been shown to mediate the uptake of Mn in vitro [22,23,24,25]. Inactivation of ZIP8 causes severe Mn deficiency [19,20,26], whereas loss-of-function mutations in ZIP14 result in hyperaccumulation of Mn in the blood and brain of affected individuals [21,27,28,29,30]. These contrasting phenotypes reflect the different functions of ZIP8 and ZIP14 in the control of systemic Mn homeostasis. ZIP14 is localized at the basolateral membranes of epithelial cells [22,31]. ZIP14 is required for the restriction of dietary Mn absorption by enterocytes [32] and the clearance of excess Mn from the portal blood by hepatocytes [33]. ZIP8 is localized at the apical canalicular membrane of hepatocytes where it is required for the reclamation of Mn from the bile [34]. Thus, while ZIP14 serves in preventing excess accumulation of Mn in the systemic circulation, ZIP8 ensures stable systemic Mn levels when Mn absorption from dietary sources is low.

Suggestive of an important function for ZIP8 in the Mn metabolism of the lungs, ZIP8 is by far most abundantly expressed in lung tissue in humans [25,35]. On the contrary, ZIP14 is expressed at rather low levels in the lungs compared to other tissues. To investigate the contribution of both transporters in Mn accumulation by alveolar epithelial cells, we used A549 cells, a well-established model for Type II alveolar cells [36]. We initially hypothesized that Mn uptake by these cells is primarily mediated by ZIP8 with only minor contributions of ZIP14. Unexpectedly, we found that, despite the higher expression of ZIP8 compared to ZIP14 in A549 cells, similar amounts of ZIP8 and ZIP14 are present at the plasma membrane. Consistently, siRNA-mediated knockdown of ZIP8 and ZIP14 revealed that both transporters contribute significantly to Mn uptake by A549 cells. Overall, our results identify ZIP8 and ZIP14 as the major transporters mediating Mn uptake by A549 cells.

## 2. Materials and Methods

### 2.1. Cell Cultures

All cell lines were obtained from the American Type Culture Collection (ATCC, Manassas, VA, USA). The A549 (ATCC CCL-185), HEK293 (ATCC CRL-1573), CaCo-2 (ATCC HTB-37), and HepG2 (ATCC HB-8065) cells were maintained at 37 °C and 5% CO_2_ in the humidified atmosphere of an incubator. The growth medium was 90% Dulbecco’s modified Eagle medium (DMEM) containing 1 mM pyruvate (Corning, Corning, NY, USA) supplemented with 3.7 g × L^−1^ NaHCO_3_, 100 units × mL^−1^ penicillin, 100 μg × mL^−1^ streptomycin (Thermo Fisher Scientific, Waltham, MA, USA), and 10% fetal bovine serum (VWR, Radnor, PA, USA) for A549 and HEK293 cells, 80% DMEM containing 1 mM pyruvate supplemented with 3.7 g × L^−1^ NaHCO_3_, 1× non-essential amino acids (Thermo Fisher Scientific, Waltham, MA, USA), 100 units × mL^−1^ penicillin, 100 μg × mL^−1^ streptomycin and 20% fetal bovine serum for CaCo-2 cells, and Eagle’s Minimum Essential Medium with L-glutamine (LONZA, Walkersville, MD, USA) supplemented with 100 units × mL^−1^ penicillin, 100 μg × mL^−1^ streptomycin, and 10% fetal bovine serum for HepG2 cells. The cells were split every three days following detachment by treatment with 0.25% trypsin in phosphate-buffered saline (PBS) without calcium and magnesium and containing 0.2% EDTA (Thermo Fisher Scientific). For routine cell culture, the cells were seeded in cell culture dishes of 55 cm^2^ (Thermo Fisher Scientific) at a density of 0.07 × 10^4^ (A549 cells), 0.18 × 10^4^ (HEK293 cells), 0.1 × 10^4^ (CaCo-2 cells) or 0.18 × 10^4^ (HepG2 cells), or in wells of a 6-well plate with a surface area of 9 cm^2^ at a density of 0.09 × 10^4^ (A549 cells), 0.11 × 10^4^ (HEK293 cells), 0.11 × 10^4^ (CaCo-2 cells) or 0.17 × 10^4^ (HepG2 cells).

### 2.2. RNAi-Mediated Knock-Down of ZIP8 and ZIP14

Lipofectamine RNAiMAX transfection reagent (Invitrogen, Carlsbas, CA, USA) was used to transfect siRNA (Origene, Rockville, MD, USA) into A549 cells. Briefly, 3.75 μL of Lipofectamine RNAiMAX and 20 pmol of scrambled siRNA, 20 pmol ZIP14-siRNA (5‘-CCC UCU GGA AGA UUA UUA UGU CUC C-3‘), 8.3 pmol ZIP8-siRNA (5‘-CCC AAA CUG UCA GAA AUA GGG ACG A-3‘), or 20 pmol ZIP14-siRNA and 8.3 pmol ZIP8-siRNA were mixed in 250 μL DMEM containing 1 mM pyruvate, incubated at room temperature (RT) for 15 min, and added into a well of a 6 well plate. A suspension of A549 cells with a density of 0.55 × 10^5^ cells per mL was prepared in A549 culture medium. In addition, 2 mL of the cell suspension were mixed with the transfection mixture, and the cells were incubated for 72 h. Successful knockdown of ZIP8 and ZIP14 was confirmed by immunoblot analysis.

### 2.3. Antibodies

To detect ZIP14 in cells, we used the rabbit anti-human ZIP14 (hZIP14) antibody described previously [32]. The observation of monomeric and multimeric forms of hZIP14 with this antibody depends on the sample preparation (Figure S1), impeding any conclusion on the ZIP14-species present in living cells. The rabbit anti-ZIP8 polyclonal antibody (20459-1-AP), horseradish peroxidase (HRP)-conjugated mouse anti-beta ACTIN monoclonal antibody (HRP-60008), HRP-conjugated mouse anti-GAPDH monoclonal antibody (HRP-60004), HRP-conjugated mouse anti-alpha Tubulin (HRP-66031), and HRP-conjugated goat anti mouse IgG (H + L) secondary antibody (SA00001-1) were from Proteintech, Rosemont, IL, USA. The goat anti-transferrin receptor (TfR) polyclonal antibody and the HRP-conjugated rabbit anti goat IgG (H + L) secondary antibody (HAF017) were from R & D systems, Minneapolis, MN, USA.

### 2.4. Real-Time PCR

Total RNA was isolated from A549, CaCo-2, HEK293, and HepG2 cells using the Quick-RNA MiniPrep Plus kit (Zymo Research, Irvine, CA, USA). First strand cDNA was synthesized from the isolated RNA using M-MuLV Reverse Transcriptase (New England Biolabs, Ipswich, MA, USA). Quantitative real-time PCR was performed using SYBR Green PCR Master-Mix (Thermo Fisher) and an Applied Biosystems QuantStudio 5 real time PCR system. The primers used for ZIP8 were (forward, 5‘-TGG TTG CAC CCC TCA CAA AT-3‘ and reverse, 5‘-CAC ATG GTG CAC TGA AAC CG-3‘). The primers used for ZIP14 (forward, 5‘-CTG GAC CAC ATG ATT CCT CAG-3‘ and reverse, 5‘-GAG TAG CGG ACA CCT TTC AG-3‘) were designed to target all known variants of ZIP14. Copy numbers of ZIP8 and ZIP14 mRNA were calculated by comparing C_t_ values with those obtained from standard curves. The PCR-product of the primers used for ZIP8 and the plasmid pCMV-Entry-hZIP14-myc-FLAG were used as standards for ZIP8 and ZIP14, respectively.

### 2.5. Immunoblot Analysis

Immunoblot analysis was performed as described previously [32]. To quantify the specific contents of ZIP8 and ZIP14, standards were prepared in NETT buffer (150 mM NaCl, 5 mM EDTA, 10 mM Tris, 1% Triton X-100 and 1× protease inhibitor cocktail (Bimake, Houston, TX, USA), pH 7.4) from the immunogens used to generate the rabbit anti-hZIP8 antibody (SLC39A8 Fusion Protein, Ag14292, Proteintech) and rabbit anti-hZIP14 antibody (GST-N-terminal hZIP14 Fusion Protein [32]), respectively.

### 2.6. Cellular Fractionation

The A549 cells were grown for three days on cell culture dishes of 55 cm^2^ and 30 × 10^6^ cells were processed with the Minute^TM^ Plasma Membrane Protein Isolation Cell Fractionation Kit (Invent Biotechnologies, Plymouth, MN, USA) according to the supplier’s instructions. The obtained fractions were mixed with 1× Sample Buffer (1.7% (*w*/*v*) SDS, 5% (*v*/*v*) glycerol, 58 mM Tris, pH 6.8), sonicated with 10 strikes (2 s, amplitude 20) of a Q55 sonicator (Qsonica L.L.C, Newton, CT, USA) and stored at −80 °C for later immunoblot analysis.

### 2.7. Cell Surface Biotinylation

Cell surface biotinylation of A549 cells grown for three days on cell culture dishes of 55 cm^2^ was performed as described previously for CaCo-2 cells [32].

### 2.8. ICP-MS Analysis of Metals

The inductively coupled plasma mass spectrometry (ICP-MS) analysis of A549 cells grown for three days in cell culture dishes of 55 cm^2^ was performed as described previously [32]. The digested samples were analyzed at the Arizona Laboratory for Emerging Contaminants (ALEC), Tucson, AZ, USA.

### 2.9. Preparation of Radiolabeled Metal Solutions

Radiolabeled metal solutions were prepared on the day of the experiment. Two-times concentrated (0.2 μM) radiolabeled Mn solutions were prepared from ^54^MnCl_2_ (PerkinElmer Inc., Waltham, MA, USA) complexed to citrate prior to addition to DMEM. Higher concentrations of Mn were achieved by adding Mn-citrate in the required amounts.

### 2.10. Metal Uptake

The Mn uptake was studied in A549 cells grown on 6 well plates for 72 h. During the experiments, cells were incubated at 37 °C (4 °C) in the humidified atmosphere of an incubator (on ice) and all solutions required before the termination of the experiment were pre-warmed (pre-chilled) to 37 °C (4 °C). At the start of the experiments, cells were washed twice with 2 mL PBS^Ca/Mg^. If not stated otherwise, 1 mL transport medium (DMEM containing 1 mM pyruvate supplemented with 20 mM HEPES, pH 7.4) was added and the cells were preincubated for 30 min. Metal uptake experiments were initiated by adding 1 mL of a freshly prepared two-times concentrated radiolabeled metal solution and the cells were incubated for the desired times. The experiments were stopped by aspirating the media and washing the cells three times with ice-cold PBS supplemented with 1 mM EDTA. The cells were lysed in 1 mL 0.5 M NaOH and an aliquot of 100 μL was used to determine the cellular protein content according to the Lowry method [37] using bovine serum albumin (BSA) as a standard. An aliquot of 200 μL to 800 μL of the lysate were used to quantify the cellular content of ^54^Mn by γ-counting.

### 2.11. Statistical Analysis

Significance of differences among two sets of data were analyzed using the Student’s *t*-test. Comparisons between multiple sets of data were performed using one- or two-way analysis of variance (ANOVA) followed by the Bonferroni post-hoc test, with * *p* < 0.05, ** *p* < 0.01, and *** *p* < 0.001. *p* > 0.05 was considered as not significant. The PRISM 5 software (GraphPad, La Jolla, CA, USA) was used for statistical analysis.

## 3. Results

### 3.1. A549 Cells Express Higher Levels of ZIP8 than ZIP14

**Figure 1:**
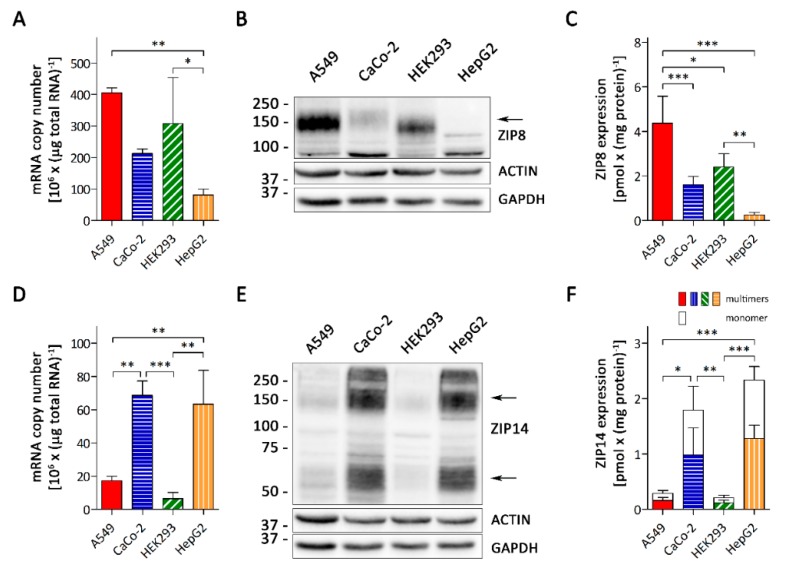
Expression of ZIP8 and ZIP14 in human cell lines. (**A**) ZIP8 and (**D**) ZIP14 mRNA copy number of A549, CaCo-2, HEK293, and HepG2 cells. Data are presented as means ± SD from three independent experiments. Statistical analysis was performed using one-way ANOVA followed by the Bonferroni post-hoc test with * *p* < 0.05, ** *p* < 0.01, and *** *p* < 0.001. (**B**) ZIP8 and (**E**) ZIP14 immunoblot of whole-cell lysates of A549, CaCo-2, HEK293, and HepG2 cells. Both β-ACTIN and GAPDH were used as loading controls. Specific bands for ZIP8 and ZIP14 (monomers and multimers) are indicated by arrows. Specific amounts of (**C**) ZIP8 and (**F**) ZIP14 (monomeric + multimeric forms) in A549, CaCo-2, HEK293, and HepG2 were determined using the fusion proteins that were used for the generation of the ZIP8- and ZIP14-antibodies as quantitative markers (see Figure S3 for details). Data are presented as means ± SD from four independent experiments. Statistical analysis was performed using one-way ANOVA followed by the Bonferroni post-hoc test with * *p* < 0.05, ** *p* < 0.01, and *** *p* < 0.001.

The alveolar epithelium consists of Type I and Type II cells [17]. As these cells may differ in their transporter expression, we first assessed whether A549 cells, a Type II alveolar cell line, provided a suitable model to study the function of ZIP8 and ZIP14 in the alveolar epithelia. In humans, ZIP8 is most abundantly expressed in the lungs and at a much lower levels in the intestine, kidney, and liver, whereas ZIP14 expression levels are highest in the liver and intestine but very low in the lungs [25,35]. We compared the mRNA and protein levels of ZIP8 and ZIP14 in A549 cells with those in CaCo-2, HEK293, and HepG2 cells that we used as cell models for the intestine, kidney, and liver, respectively. Real-time PCR and immunoblot analyses revealed that A549 cells expressed the highest amounts of ZIP8 among the cell lines tested, at both mRNA and protein levels (Figure 1A–C and Figure S2A,B). In contrast, A549 cells contained only low levels of ZIP14 mRNA and protein compared to CaCo-2 and HepG2 cells (Figure 1D–F and Figure S2C,D).

The expression profile of both ZIP8 and ZIP14 in A549, CaCo-2, HEK293, and HepG2 cells reflects the organ-specific expression in vivo. Thus, A549 cells can be regarded as a valid model to study the functions of ZIP8 and ZIP14 in the alveolar epithelia.

In A549 cells, the copy number of ZIP8 mRNA were about 23 times that of ZIP14 mRNA (Figure 1A,D), suggesting that ZIP8 was strongly enriched over ZIP14 in these cells. To allow for a direct comparison of ZIP8 and ZIP14 protein levels, we determined the specific amounts of both transporters employing the fusion-peptides used to generate the respective antibodies as quantitative markers (Figure S3). Consistent with the outcome from the real-time PCR analysis, the specific amounts of ZIP8 were found to be about 14 times that of ZIP14 (Figure 1C,F).

### 3.2. Characterization of Mn Accumulation by A549 Cells

**Figure 2:**
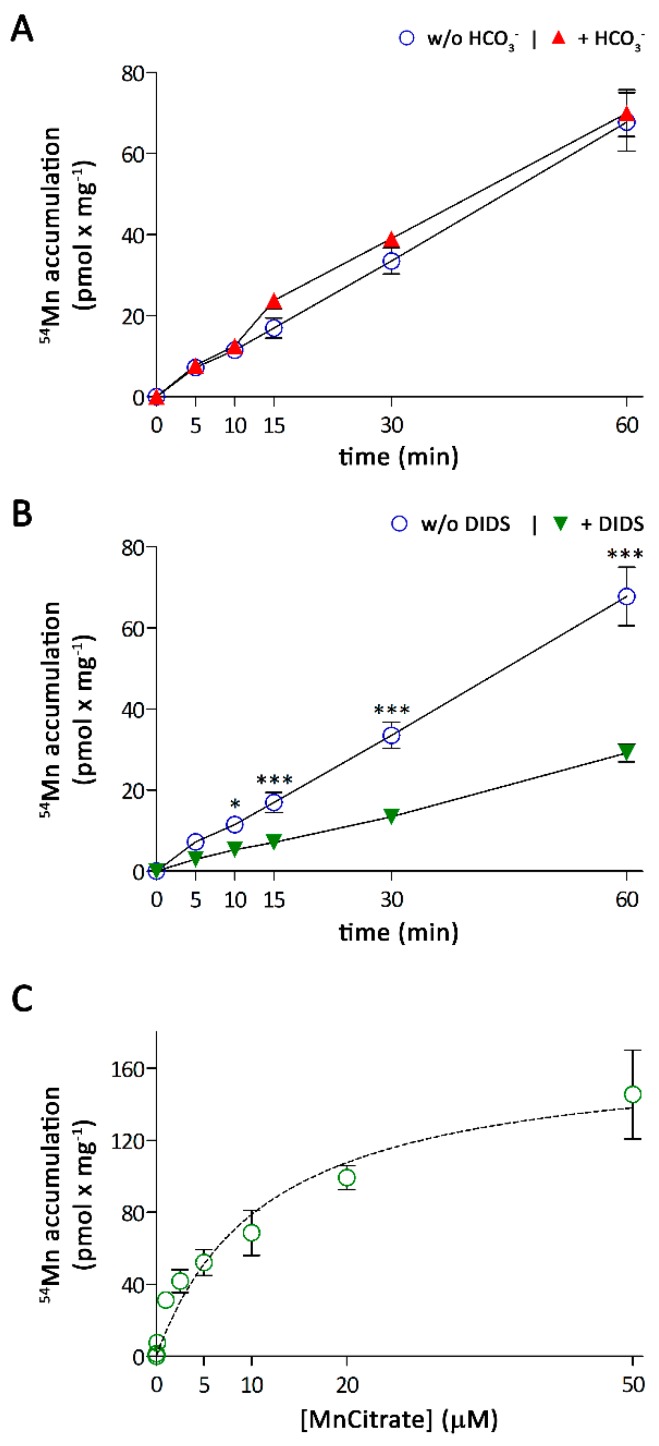
Characterization of ^54^Mn accumulation by A549 cells. (**A**) Dependence of ^54^Mn accumulation on exogenous HCO_3_^−^. (**B**) Effect of 1 mM 4,4-thiocyanostilbene-2,2-disulfonic acid (DIDS) on ^54^Mn accumulation. (**C**) Concentration-dependence of ^54^Mn accumulation. Data are presented as means ± SD from three independent experiments performed in duplicates. Statistical analysis was performed using (**A**,**B**) two-way ANOVA followed by the Bonferroni post-hoc test with * *p* < 0.05 and *** *p* < 0.001.

Because both ZIP8 and ZIP14 are bicarbonate-dependent transporters [22,23,24], we next tested the effect of bicarbonate (HCO_3_^−^) on ^54^Mn accumulation. We incubated A549 cells for up to 60 min with 0.1 μM ^54^Mn in DMEM^B^ (DMEM, 1 mM pyruvate 25 mM HEPES, 44 mM NaHCO_3_, pH 7.4) or DMEM^H^ (DMEM, 1 mM pyruvate, 25 mM HEPES, pH 7.4) at 10% CO_2_ and 0% CO_2_, respectively. The A549 cells readily accumulated ^54^Mn irrespective of the experimental medium (Figure 2A). However, in the presence of the anion exchanger inhibitor 4,4-thiocyanostilbene-2,2-disulfonic acid (DIDS), ^54^Mn accumulation was reduced by more than 60% (Figure 2B), suggesting that anion exchange facilitates Mn uptake by A549 cells. Since DMEM^H^ contains HCO_3_^−^ derived from dissolved CO_2_ contained in the air and CO_2_/HCO_3_^−^ released by the cells, we concluded that this low concentration of HCO3^−^ was already sufficient to support Mn uptake by endogenous ZIP8 and/or ZIP14. Thus, if not stated otherwise, we used DMEM^H^ for all further experiments.

The ZIP8- and ZIP14-mediated Mn uptakes have been previously described to follow Michaelis–Menten kinetics, with a K_M_-value of 2.2 ± 0.3 μM for ZIP8 [23] and K_M_-values of 4.4 ± 0.5 μM and 18.2 ± 2 μM for the isoforms ZIP14A and ZIP14B, respectively [22]. To characterize the kinetics of Mn accumulation by A549 cells, we measured the initial rates of ^54^Mn accumulation at five minutes using a wide range of Mn concentrations (Figure 2C). The ^54^Mn accumulation by these cells followed Michaelis–Menten kinetics with an apparent K_M_ of 12 ± 2 μM, which falls between the K_M_ values reported for ZIP8 and ZIP14A, the most abundant ZIP14 isoform in human lung tissue [21].

**Figure 3:**
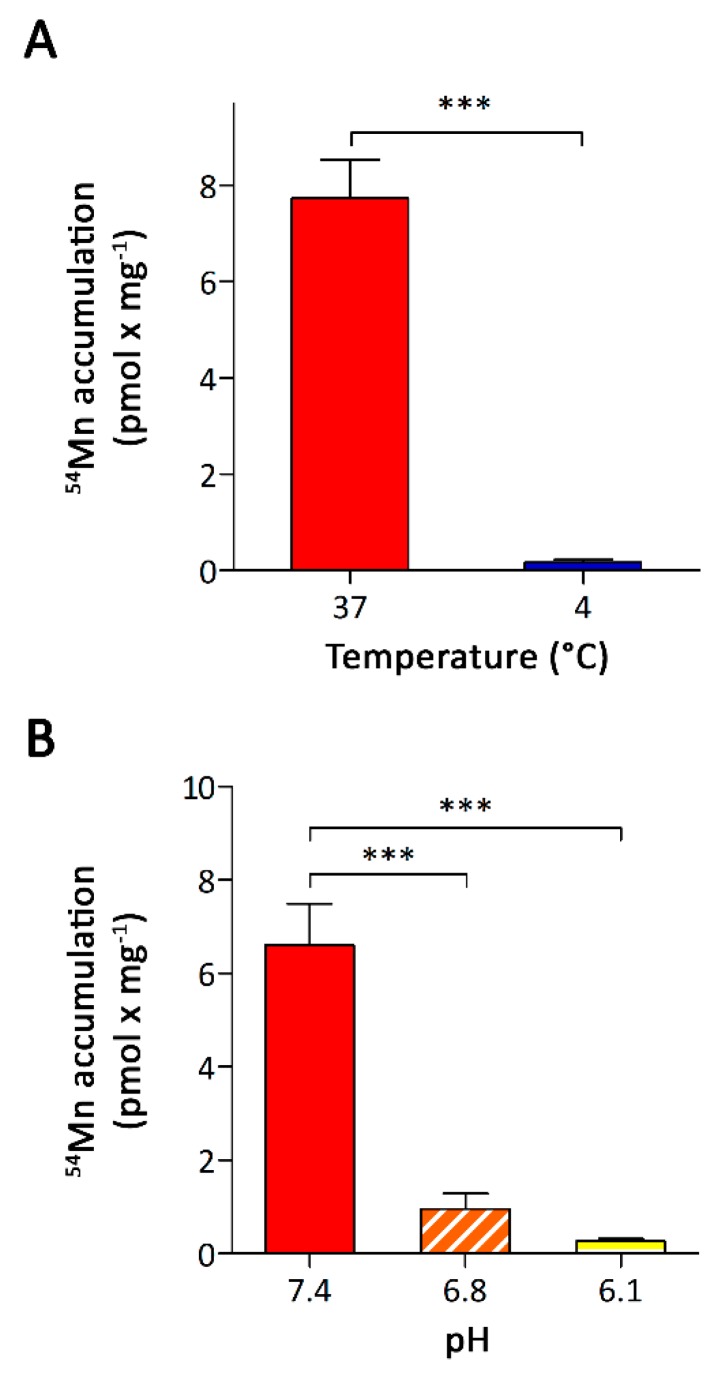
Effects of temperature and extracellular pH on ^54^Mn accumulation by A549 cells. (**A**) Temperature dependence of ^54^Mn accumulation and (**B**) dependence of ^54^Mn accumulation on the extracellular pH. Data are presented as means ± SD from three independent experiments performed in duplicates. Statistical analysis was performed using (**A**) student’s *t*-test or (**B**) one-way ANOVA followed by the Bonferroni post-hoc test with *** *p* < 0.001.

To confirm that the accumulation of ^54^Mn by A549 cells was due to the active membrane-dependent uptake mechanism, A549 cells were incubated at 4 °C with 0.1 μM ^54^Mn for 5 min. The specific amount of accumulated ^54^Mn was significantly decreased to about 2% of that determined for cells that had been incubated at 37 °C (Figure 3A).

Next, we investigated the pH dependence of Mn accumulation by incubating A549 cells for 5 min with 0.1 μM ^54^Mn in DMEM^P^ (DMEM, 1 mM pyruvate, 25 mM PIPES) adjusted to pH 6.1, pH 6.8 or pH 7.4. We used DMEM^P^ instead of DMEM^H^, since the use of PIPES allowed us to use the same buffer for the pH-range investigated. In doing so, we avoided any potential confounding effects that could have resulted from the use of different buffering compounds. The ^54^Mn accumulation by A549 cells decreased dramatically with decreasing pH (Figure 3B), ruling out a major involvement of DMT1-mediated Mn uptake. The similar pH dependencies reported for ZIP8-mediated cadmium and iron uptake [23,25] and ZIP14-mediated Mn, iron, and zinc uptake [24,32,38] would be consistent with a role of ZIP8, ZIP14, or both in Mn accumulation by A549 cells.

### 3.3. Consequences of siRNA-Mediated Knockdown of ZIP8 and ZIP14

**Figure 4:**
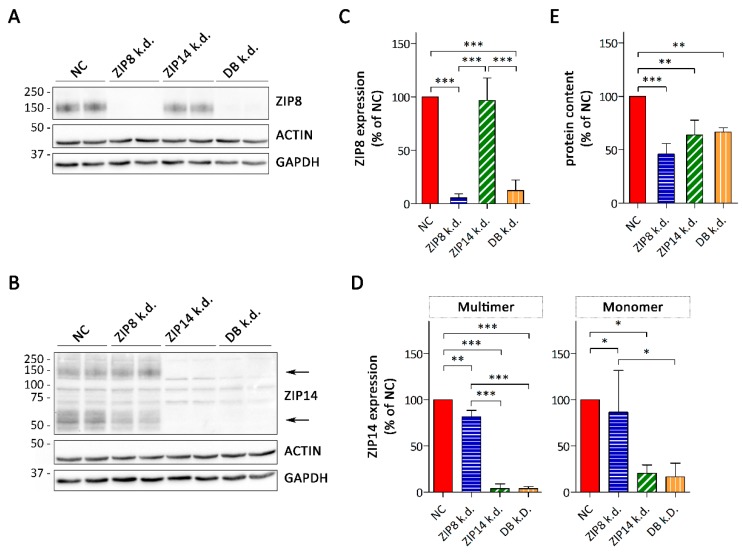
RNAi-mediated knockdown of ZIP8, ZIP14 or both in A549 cells. (**A**) ZIP8 and (**B**) ZIP14 immunoblot of whole-cell lysates confirming the knockdown of ZIP8 and ZIP14 (NC, scrambled control; DB, combined knockdown of ZIP8 and ZIP14). Both β-ACTIN and GAPDH were used as loading controls. The relative expression of (**C**) ZIP8 and (**D**) ZIP14 was determined by normalizing the band intensities to β-ACTIN. Data are presented as means ± SD from three independent experiments. Statistical analysis was performed using one-way ANOVA followed by the Bonferroni post-hoc test with ** *p* < 0.01 and *** *p* < 0.001. (**E**) Protein contents of A549 cultures. Data are presented as means ± SD from three independent experiments performed in duplicates. Statistical analysis was performed using one-way ANOVA followed by the Bonferroni post-hoc test with * *p* < 0.05, ** *p* < 0.01, and *** *p* < 0.001.

To specifically assess the contributions of ZIP8 and ZIP14 to Mn accumulation by A549, we used siRNA technology to suppress the expression of ZIP8 and ZIP14, individually or in combination. Successful ZIP8- and ZIP14-knockdown was confirmed by immunoblot analysis. When ZIP8 was knocked down individually, ZIP8 protein levels decreased to below 10% of that in scramble siRNA-treated controls (Figure 4A,B and Figure S4A,B). In the combined knockdown, the expression levels of ZIP8 were slightly higher compared to the single knockdown of ZIP8, but still remained below 15% of the control. Single knockdown of ZIP14 resulted in a decrease in multimeric and monomeric forms of ZIP14 to below 10% and 20% of the controls, respectively (Figure 4C,D and Figure S4C,D). The expression level of ZIP14 in the combined knockdown did not differ from that in the single ZIP14 knockdown.

Microscopic observation of ZIP8- and/or ZIP14-silenced A549 cells revealed a reduction in cell numbers compared to the control, and protein contents in these cells were significantly lower (Figure 4E). Since the cell density correlated with the total protein content of A549 cultures (Figure S5) and no obvious signs of toxicity were observed, this result suggests that knockdown of ZIP8- and/or ZIP14 impairs proliferation of A549 cells. Knockdown of ZIP8 has recently been shown to inhibit the proliferation of SH-SY5Y cells, a human-derived neuroblastoma cell line, through induction of cell cycle arrest [39] and of primary myoblasts [40]. In mice, knockout of ZIP14 causes a decrease in hepatocyte proliferation [41]. Thus, our observations in A549 cells reinforce a potential, critical role of both ZIP8 and ZIP14 in the proliferation of mammalian cells.

**Figure 5:**
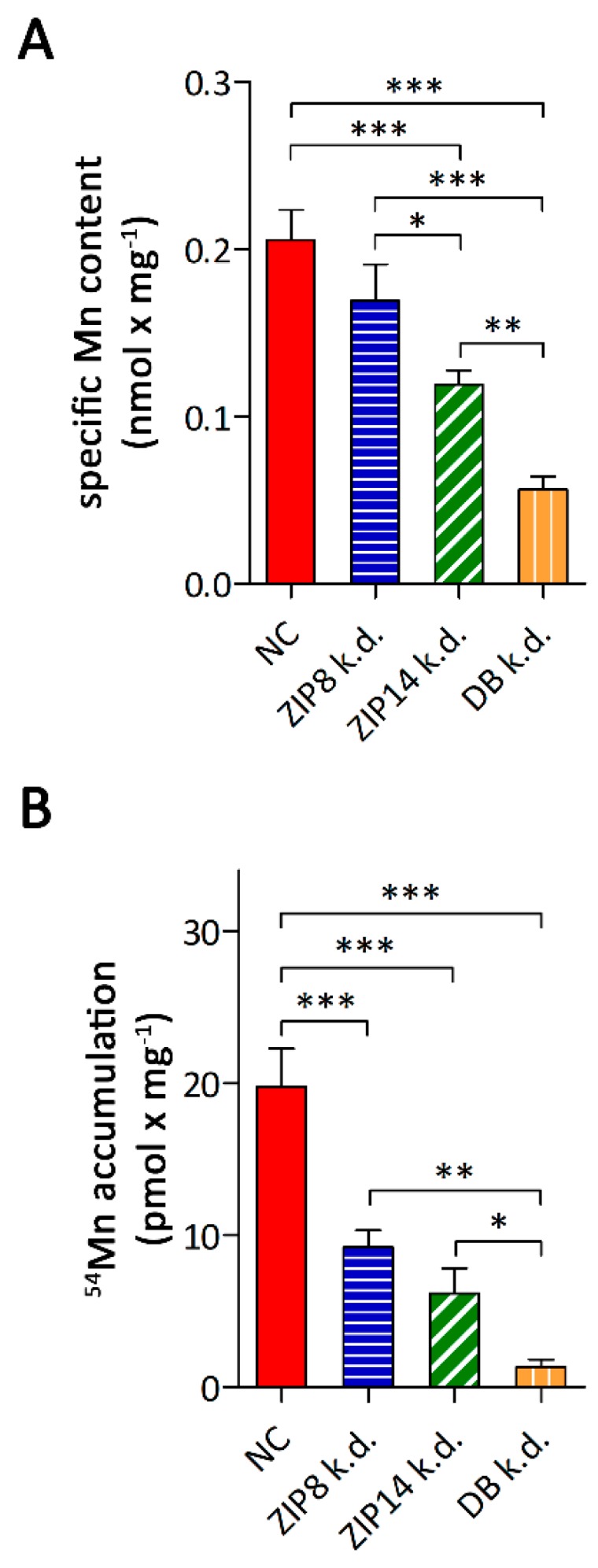
Consequences of RNAi-mediated knockdown of ZIP8 and/or ZIP14 on Mn metabolism of A549 cells. (**A**) specific Mn contents and (**B**) ^54^Mn accumulation. Data are presented as means ± SD from three independent experiments performed in duplicates. Statistical analysis was performed using one-way ANOVA followed by the Bonferroni post-hoc test with * *p* < 0.05, ** *p* < 0.01, and *** *p* < 0.001.

Next, we studied the consequences of ZIP8- and/or ZIP14-knockdown on the specific Mn contents and ^54^Mn accumulation. Knockdown of ZIP14 as well as combined knockdown of ZIP8 and ZIP14 significantly lowered the specific Mn contents of A549 cells to 58% and 28% of the controls, respectively (Figure 5A). In A549 cells knocked down in ZIP8 alone, the specific Mn contents, albeit trending to be lower, did not differ significantly from control cells. However, the lower Mn levels observed in A549 cells upon combined knockdown of ZIP8 and ZIP14 compared to single ZIP14 knockdown, indicate that both ZIP8 and ZIP14 contribute to Mn metabolism of A549 cells. In support of this view, knockdown of ZIP8 reduced ^54^Mn accumulation by 50% and silencing of ZIP14 caused a decrease by 70% compared to the controls (Figure 5B). The combined knockdown of ZIP8 and ZIP14 caused a reduction of ^54^Mn accumulation by 94% of the control, suggesting that ZIP8 and ZIP14 are the major transporters mediating Mn accumulation by A549 cells.

To rule out that the decreased Mn accumulation by A549 cells upon knockdown of ZIP8 and/or ZIP14 was a consequence of the impaired proliferation of these cells, we knocked down both transporters individually or in combination in HepG2 cells (Figure S6A–E). Similar to A549 cells, knockdown of ZIP8 and/or ZIP14 resulted in impaired proliferation of HepG2 cells (Figure S6F). However, consistent with the low expression levels of ZIP8 in HepG2 cells, Mn accumulation was only affected by knockdown of ZIP14, but not by knockdown of ZIP8 (Figure S6G).

### 3.4. Sucellular Localization of ZIP8 and ZIP14 in A549 Cells

Although the expression of ZIP8 in A549 cells was about 14 times higher than that of ZIP14 (Figure 1A,C,D,F), knockdown of ZIP8 or ZIP14 resulted in similar decreases in Mn accumulation (Figure 5B). This could either reflect differences in the maximum transport rate (V_max_) for Mn or similar cell surface levels of ZIP8 and ZIP14. Hence, we next studied the cellular localization of ZIP8 and ZIP14 in A549 cells by two independent methods.

**Figure 6:**
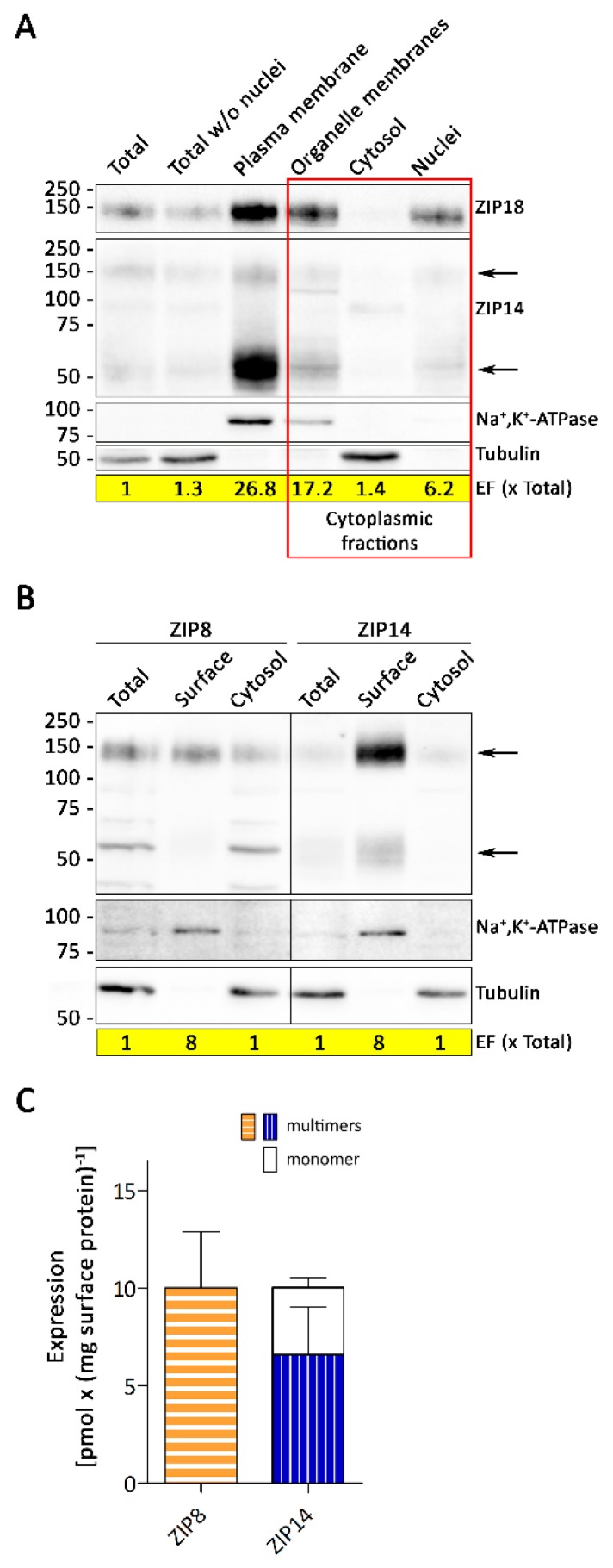
Subcellular localization of ZIP8 and ZIP14 in A549 cells. (**A**) Plasma membranes and three cytoplasmic fractions were isolated from whole-cell lysates (Total); 40 μg of each fraction was analyzed by immunoblotting for ZIP8 and ZIP14. The fractions were enriched compared to the whole-cell lysate by the enrichment factors (EF) indicated. Na^+^, K^+^-ATPase, and Tubulin served as plasma membrane and cytosolic markers, respectively. (**B**,**C**) A549 cultures were subjected to surface biotinylation with cell membrane impermeable Sulfo-NHS-SS-biotin after which the cells were lysed in 1000 μL NETT buffer. Biotin-labeled cell-surface proteins were isolated from 800 μL whole-cell lysates using NeutrAvidin agarose beads and eluted in 100 μL sample buffer. (**B**) 40 μL of whole-cell lysates (Total), biotin-labeled cell-surface proteins (Surface) and flow-through (Cytosol) were analyzed by immunoblotting for ZIP8 and ZIP14. Cell surface proteins were enriched compared to whole-cell lysates by a factor of 8. Na^+^, K^+^-ATPase, and Tubulin served as the surface and cytosolic markers, respectively. (**C**) Specific amounts of membrane localized ZIP8 and ZIP14 (monomeric + multimeric forms) were determined using the fusion proteins that were used for the generation of the ZIP8- and ZIP14-antibodies as quantitative markers (see Figure S9 for details). Data are presented as means ± SD from five independent prepared cultures. Statistical analysis was performed using student’s *t*-test.

First, we isolated plasma membranes and three cytoplasmic fractions (cellular organelles, cytosol, and nuclei) from A549 cells and analyzed them for ZIP8 and ZIP14 (Figure 6A and Figure S7). While we detected strong signals for ZIP8 in the plasma membrane fraction as well as in the fractions containing cellular organelles and nuclei; ZIP14 was primarily confined to the plasma membrane. Second, using a biotinylation approach we discovered that ZIP8, in contrast to ZIP14, was not enriched at the plasma membrane of A549 cells (Figure 6B and Figure S8). These observations demonstrate that ZIP8 mainly localizes to intracellular compartments, whereas ZIP14 is strongly enriched at the plasma membrane of A549. Moreover, the quantification of cell surface localized ZIP8 and ZIP14 confirmed that, in spite of ZIP8 being much more abundant in whole cell-lysates than ZIP14, expression levels of both proteins at the plasma membrane are similar (Figure 6C and Figure S9).

## 4. Discussion

In the present study, we identified both ZIP8 and ZIP14 as the major transporters mediating Mn accumulation by A549 cells, a Type II alveolar epithelial cell line. Moreover, we have determined for the first time the expression levels of ZIP8 and ZIP14 in different cell lines at both mRNA and protein levels.

Recently, ZIP8 and ZIP14 have been identified as critical Mn transporters in vivo [19,20,21]. In mammalian tissues, ZIP8 is most abundantly expressed in the lungs [25], whereas ZIP14 expression levels in the lungs are rather low compared to other organs [25,35]. Validating A549 cells as a model system to study the function of ZIP8 and ZIP14 in the alveolar epithelia, ZIP8 mRNA and protein are expressed at higher levels and ZIP14 mRNA and protein at lower levels in these cells than in cell models for the liver, intestine, and kidneys. This differential expression of ZIP8 and ZIP14 further points to the tissue-specific functions of these transporters.

A549 cells have previously been used as a model system to examine pathways for direct uptake of divalent Mn by the pulmonary epithelium [18]. Manganese accumulation by these cells was time-dependent and saturable. Consistent with the observation that uptake of intratracheally instilled Mn was not affected in the Belgrade rat model [18], we observed that Mn accumulation by A549 cells was severely compromised by lowering the extracellular pH, excluding DMT1, which functions optimally at acidic pH [42], as the predominant Mn transporter in the alveolar epithelia. Moreover, our data revealed that Mn accumulation by A549 cells almost completely depends on the activities of ZIP8 and ZIP14.

Considering that in A549 cells ZIP14 is far less abundantly expressed than ZIP8, the finding that both transporters contribute to Mn accumulation to a similar extent was unexpected but is consistent with the differential subcellular distributions of ZIP8 and ZIP14 in these cells. While in A549 cells the majority of ZIP8 localizes intracellularly, ZIP14 is strongly enriched at the plasma membrane. The localization of ZIP8 appears to vary between cell types [43,44]. Similar to A549 cells, in WIF-B cells endogenous ZIP8 is almost entirely associated with intracellular structures, with only a fraction of it being localized at the canalicular membrane [45]. Endogenous ZIP8 has further been reported to localize to the plasma membrane and mitochondria in the bronchial epithelial cell line BEAS-2B [46], to the plasma membrane and lysosomes in human T cells [47], and to the apical surface and nuclei of rat inner-ear hair cells [43]. Ectopically expressed ZIP8 has been observed at the plasma membrane and in early endosomes of HEK293 cells [25], and at the apical membrane of MDCK cells [23]. Endogenous ZIP14 has been shown to be strongly enriched at the plasma membrane of CaCo-2 cells [32], HepG2 cells [38], HEK293 cells [48], and WIF-B cells [45].

Our findings that ZIP8 and ZIP14 represent the major Mn importers in A549 cells is in contrast to the study by Heilig et al. [18], who found manganese accumulation by A549 cells to be inhibited by extracellular calcium, magnesium, and different calcium channel inhibitors, and concluded that TRPM7, LVGCs, and possibly other TRP channels contribute to manganese absorption by these cells. Also, Mn accumulation was not inhibited by lowering the extracellular pH in that study [18]. We attribute these discrepancies to differences in the experimental conditions, specifically the absence of calcium and magnesium in the uptake buffer used by Heilig et al. [18] to study Mn accumulation by A549 and the presence of physiologic concentrations of these divalent metals in DMEM, which served as the experimental medium in the present study. It has been reported that calcium is required for the metal transport activity of ZIP14 [24] and for Znt10-mediated Mn export [49]. In addition, the absence of calcium and magnesium may increase unspecific Mn uptake by certain calcium and magnesium channels, altering the transporter profile contributing to Mn accumulation by A549 cells.

Type II alveolar cells are polarized cells that are in contact with the submucosa and lumen which is not taken into account in the regular A549 cultures. Thus, although we have identified here ZIP8 and ZIP14 as major Mn transporters in A549 cells, the limitation of the culture system precludes any conclusion as to whether ZIP8 and ZIP14 contribute to the absorption of airborne Mn. A further limitation of our study is that A549 cells are a tumor cell line. Studies utilizing primary alveolar type II cell cultures would not only further validate the A549 cell line for studying the Mn metabolism of type II alveolar cells, but potentially reveal differences in Mn metabolism of cancerous and healthy tissues.

Quantitative RT-PCR and quantitative immunoblot analyses revealed a strong enrichment of ZIP8 over ZIP14 in A549 cells. However, the majority of ZIP8 localizes to intracellular compartments, whereas ZIP14 is strongly enriched at the cell surface, and comparable amounts of both transporters are present at the plasma membrane. This finding is in agreement with our observation that knockdown of either ZIP8 or ZIP14 causes similar decreases in Mn accumulation by A549 cells. A predominant intracellular localization of ZIP8 has been reported previously for primary human lung epithelial cells and the immortalized human bronchial epithelial cell line BEAS-2B [46]. In these cells, ZIP8 was detected at the cell surface only after stimulation with the proinflammatory cytokine TNFα. This and our finding suggest that in addition to mediating Mn uptake at the plasma membrane, ZIP8 contributes to other (intra)cellular functions in lung epithelia. Indeed, it has been reported that ZIP8 plays a role in mitochondrial Mn transport and innate immune responses [44,46,50].

## 5. Conclusions

In conclusion, we validated here the use of A549 cells for studying the functions of ZIP8 and ZIP14 in Type II alveolar cells. Using these cells, we showed that ZIP8 and ZIP14 are crucial for efficient Mn accumulation by A549 cells. Whether these transporters are involved in the uptake of airborne Mn remains to be elucidated.

## Supplementary Materials

The following are available online at https://www.mdpi.com/2072-6643/11/7/1473/s1, Figure S1: Band appearance of ZIP14 in non-reducing and reducing conditions., Figure S2: Uncropped immunoblots for Figure 1B and Figure 1E., Figure S3: Quantification of ZIP8 and ZIP14 in human cell lines., Figure S4: Uncropped immunoblots for Figure 4A and Figure 4B., Figure S5: Correlation of cell number with the total protein content in A549 cultures., Figure S6: Consequences of RNAi mediated knockdown of ZIP8, ZIP14 individually or both in HepG2 cells., Figure S7: Uncropped immunoblots for Figure 6A., Figure S8: Uncropped immunoblots for Figure 6B., Figure S9: Quantification of ZIP8 and ZIP14 at the cell-surface of A549 cells.
