# Supplementary material for: Manganese Uptake by A549 Cells is Mediated by Both ZIP8 and ZIP14
Source: Nutrients. 2019 Jun 28;11(7):1473. doi: 10.3390/nu11071473 (PMC6682971; doi:10.3390/nu11071473)
Supplement: Supplementary file 1 [file nutrients-11-01473-s001.zip › Supporting information-With Track Change.docx]

*Supplementary Materials*

**Manganese uptake by A549 cells is mediated by both ZIP8 and ZIP14**

**Ivo F. Scheiber, Neftali Ortega Alarcon and Ningning Zhao^*^**

Department of Nutritional Sciences, The University of Arizona, Tucson, AZ 85721, USA; ifscheiber@email.arizona.edu; nortegaalarcon@email.arizona.edu; zhaonn@email.arizona.edu

***** Correspondence: zhaonn@email.arizona.edu; Tel +01-520-621-9744

**List of materials included:** Figures S1–S9


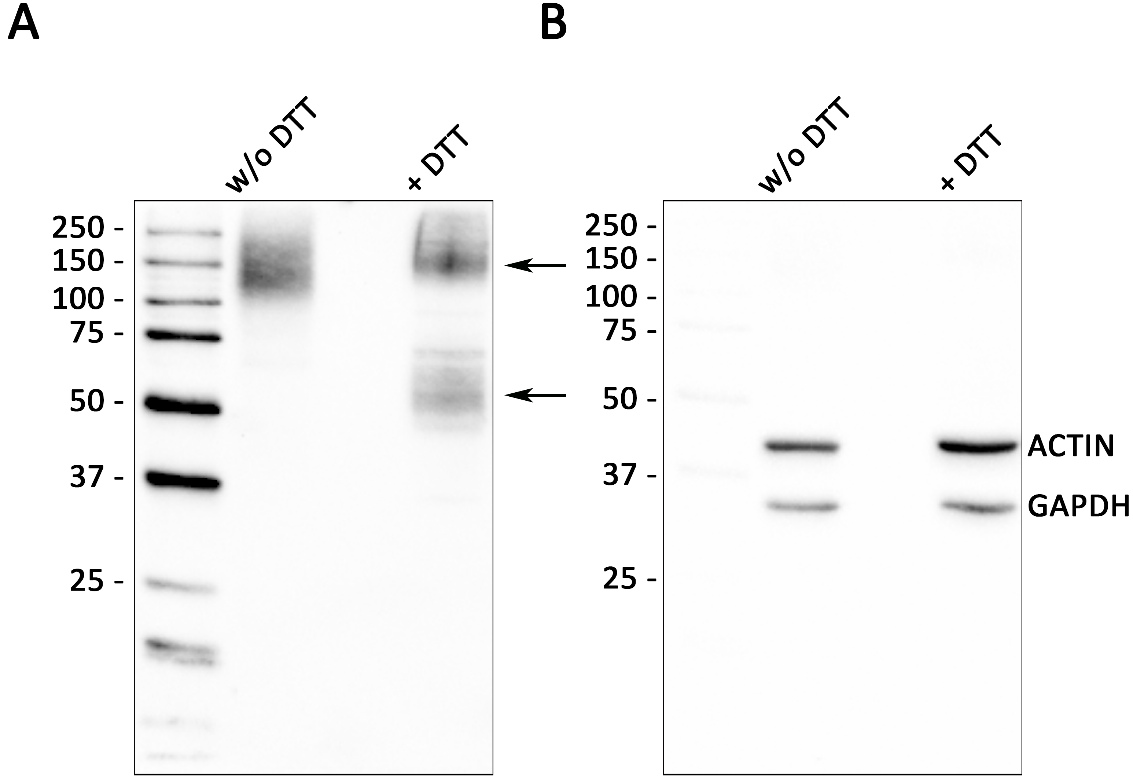


Figure S1. Band appearance of ZIP14 in non-reducing and reducing conditions. Cell lysates were mixed with 6x Laemmli buffer containing no (non-reducing condition) or 10% DTT (reducing condition) and incubated at 37°C for 30 minutes before they were analyzed by immunoblotting with anti-hZIP14 antibodies. Whole blots for (A) ZIP14 and (B) β-ACTIN and GAPDH. Specific bands for ZIP14 (monomers and multimers) are indicated by arrows. While our anti-hZIP14 detects both monomeric and multimeric forms of ZIP14 in reducing conditions, only multimeric forms can be detected when non-reducing conditions had been applied.


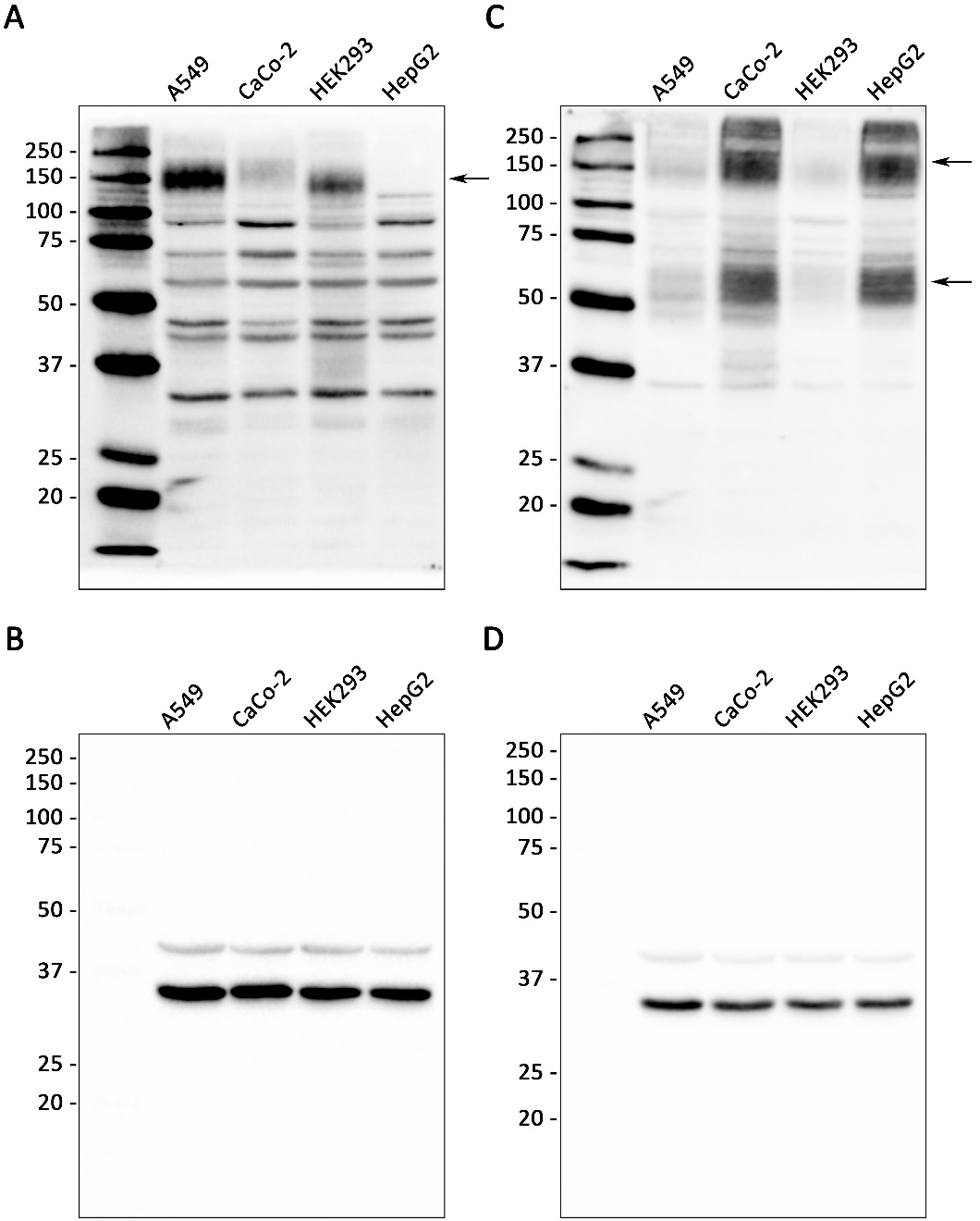


**Figure S2.** Uncropped immunoblots for Figure 1B and Figure 1E. (**A, B**) Whole blots for Figure 1B. (**A**) ZIP8, (**B**) β-ACTIN and GAPDH. (**C, D**) Whole blots for Figure 1E. (**C**) ZIP14 and (**D**) β-ACTIN and GAPDH. Specific bands for ZIP8 and ZIP14 (monomers and multimers) are indicated by arrows.
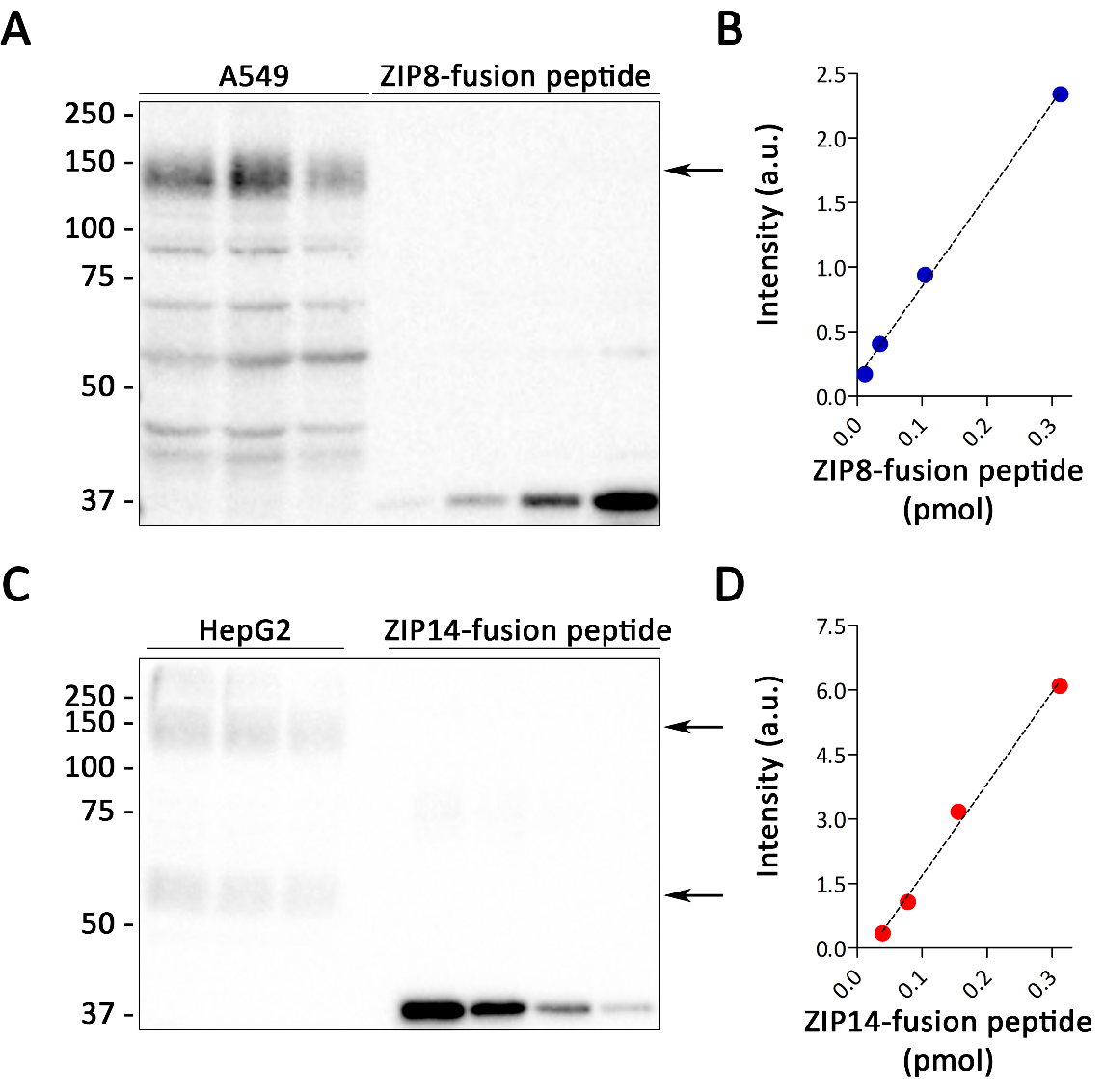
**Figure S3.** Quantification of ZIP8 and ZIP14 in human cell lines. (**A**) ZIP8 immunoblot of whole-cell lysates of A549 cells and ZIP8-fusion protein. Specific bands for ZIP8 are indicated by arrows. Band intensities were quantified by densitometric analysis. (**B**) Calibration curve for ZIP8 using the ZIP8-fusion protein as a standard. (**C**) ZIP14 immunoblot of whole-cell lysates of HepG2 cells and ZIP14-fusion protein. Specific bands for ZIP14 (monomers and multimers) are indicated by arrows. (**D**) Calibration curve for ZIP14 using the ZIP14-fusion protein as a standard. Data are from representative experiments.

**
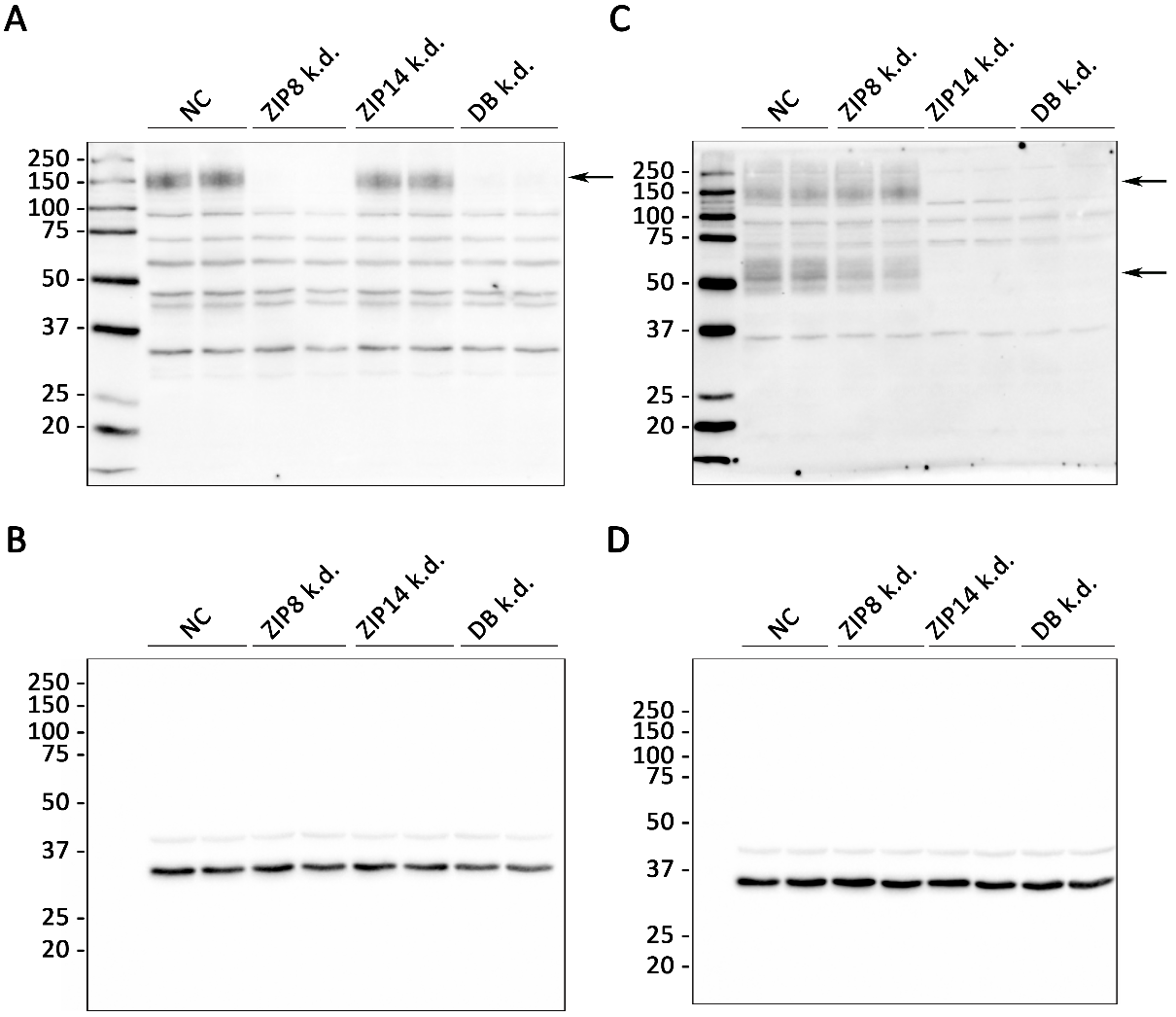
**

**Figure S4.** Uncropped immunoblots for Figure 4A and Figure 4B. (**A, B**) Whole blots for Figure 4A. (**A**) ZIP8, (**B**) β-ACTIN and GAPDH. (**C, D**) Whole blots for Figure 4B. (**C**) ZIP14 and (**D**) β-ACTIN and GAPDH. Specific bands for ZIP8 and ZIP14 (monomers and multimers) are indicated by arrows.

**
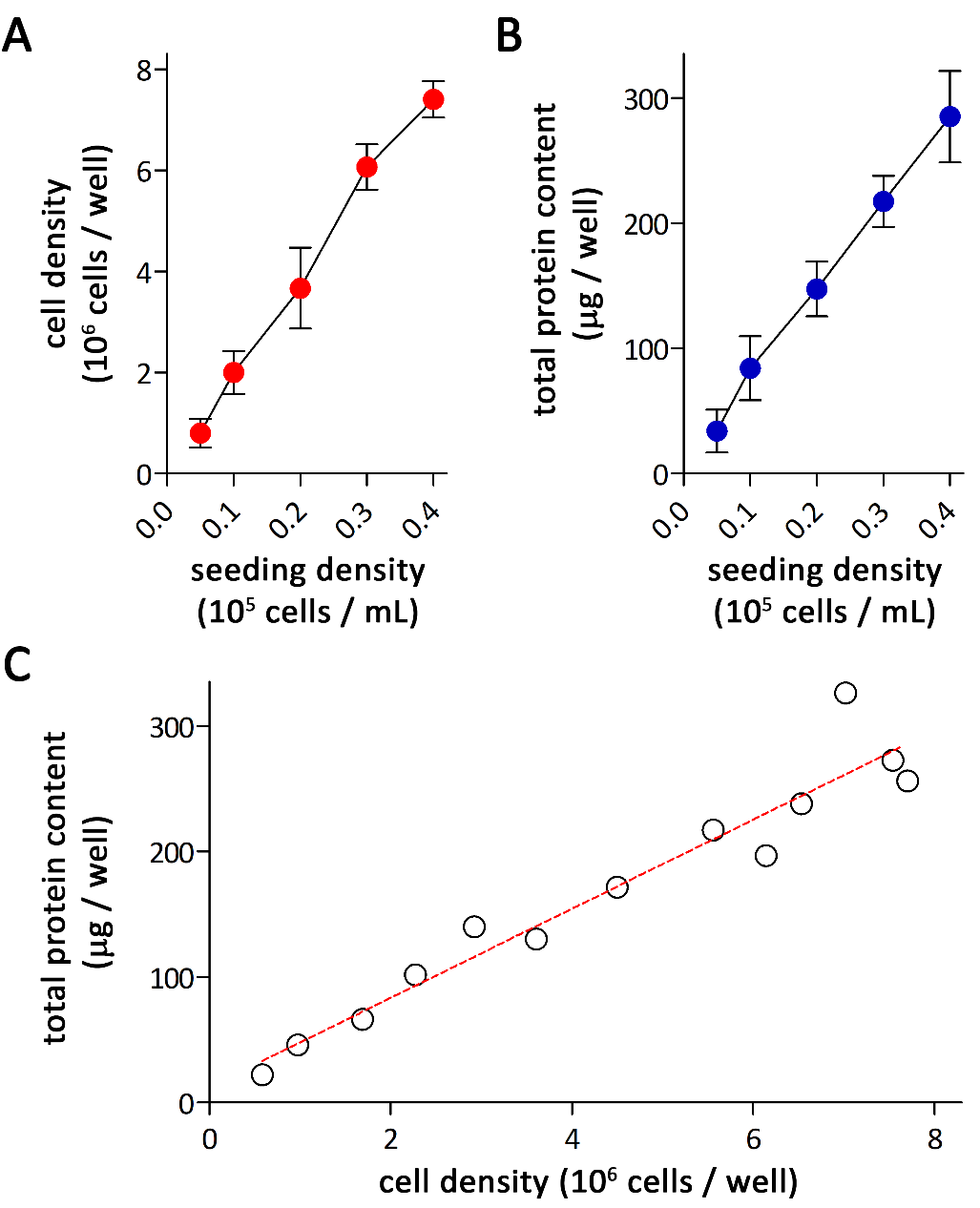
**

**Figure S5.** Correlation of cell number with the total protein content in A549 cultures. A549 cells were seeded at the cell densities indicated in panels A and B. Three days after seeding, the cells were harvested and (A) cell density and (B) total protein content were determined. Data are presented as means ± s.d. from three independent experiments. (C) Correlation of the cell density with the total protein content of the cultures.


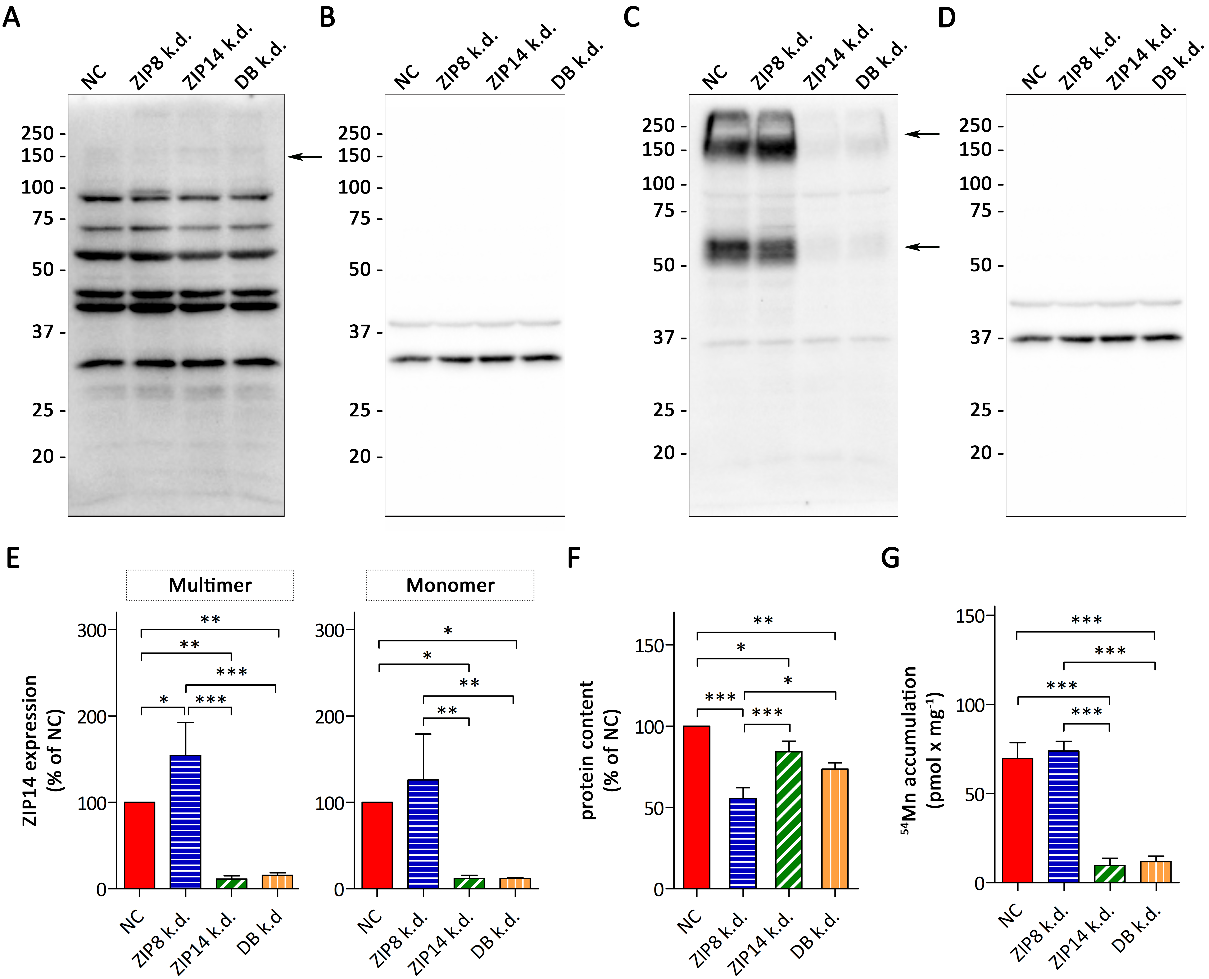


**Figure S6.** Consequences of RNAi-mediated knockdown of ZIP8, ZIP14 individually or both in HepG2 cells. (**A**) ZIP8 immunoblot of whole-cell lysates (NC, scrambled control; DB, combined knockdown of ZIP8 and ZIP14). Specific bands for ZIP8 are indicated by arrows. Equal loading was confirmed using (**B**) β-ACTIN and GAPDH as loading controls. (**C**) ZIP14 immunoblot of whole-cell lysates. Specific bands for ZIP14 (monomers and multimers) are indicated by arrows. Equal loading was confirmed using (**D**) β-ACTIN and GAPDH as loading controls. (**E**) The relative expression of ZIP14 was determined by normalizing the band intensities to β-ACTIN. Data are presented as means ± s.d. from three independent experiments. Statistical analysis was performed using one-way ANOVA followed by the Bonferroni post-hoc test with *p < 0.05, **p < 0.01 and ***p < 0.001. (**F**) Protein contents of HepG2 cultures. Data are presented as means ± s.d. from three independent experiments performed in duplicates. Statistical analysis was performed using one-way ANOVA followed by the Bonferroni post-hoc test with *p < 0.05, **p < 0.01 and ***p < 0.001. (**G**) Consequences of RNAi-mediated knockdown of ZIP8 and/or ZIP14 on ^54^Mn accumulation. Data are presented as means ± s.d. from three independent experiments performed in duplicates. Statistical analysis was performed using one-way ANOVA followed by the Bonferroni post-hoc test with ***p < 0.001.

**
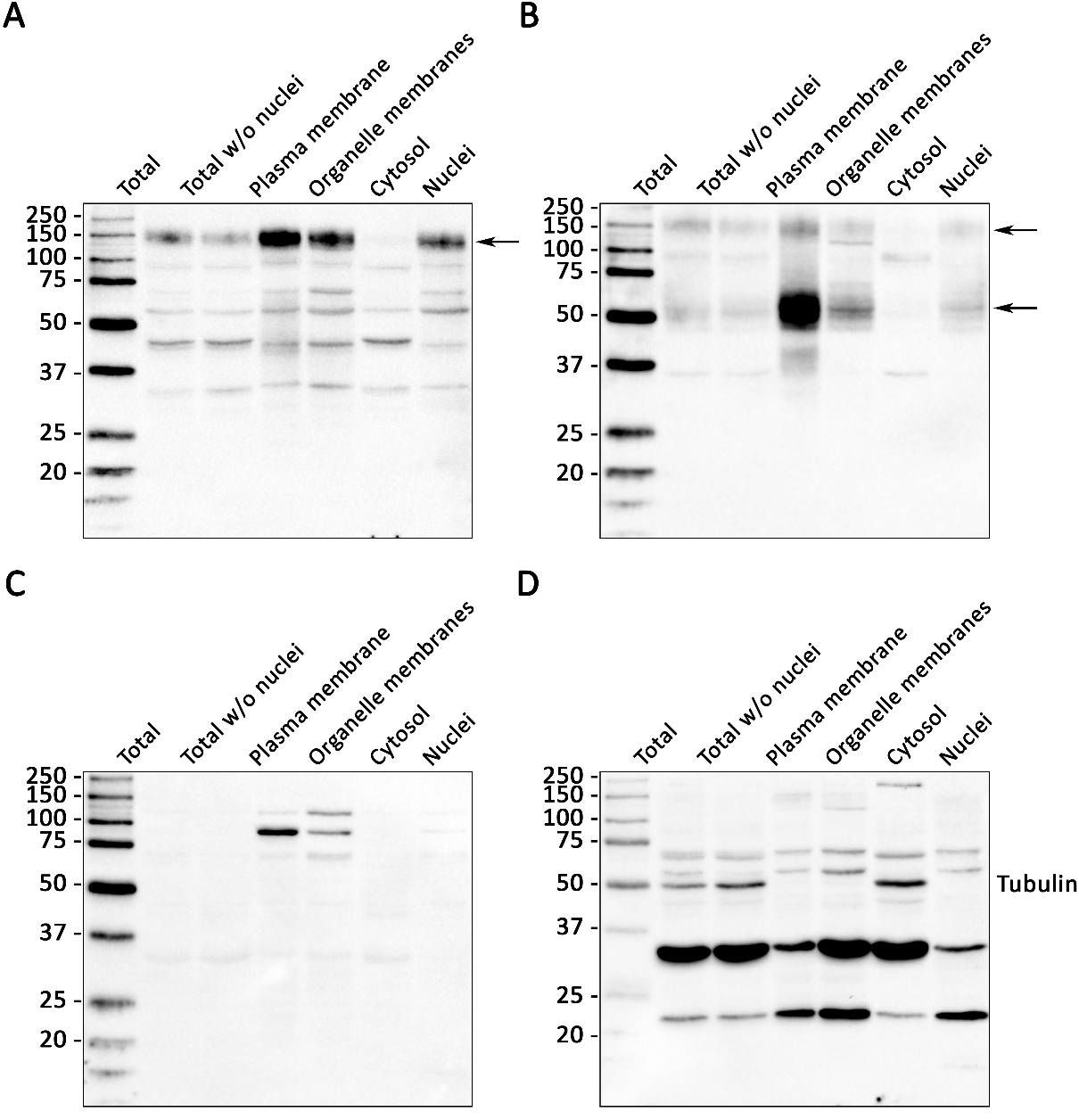
Figure S7.** Uncropped immunoblots for Figure 6A. Whole blots for (**A**) ZIP8, (**B**) ZIP14, (**C**) Na^+^, K^+^-ATPase and (**D**) Tubulin. Specific bands for ZIP8 and ZIP14 (monomers and multimers) are indicated by arrows.

**
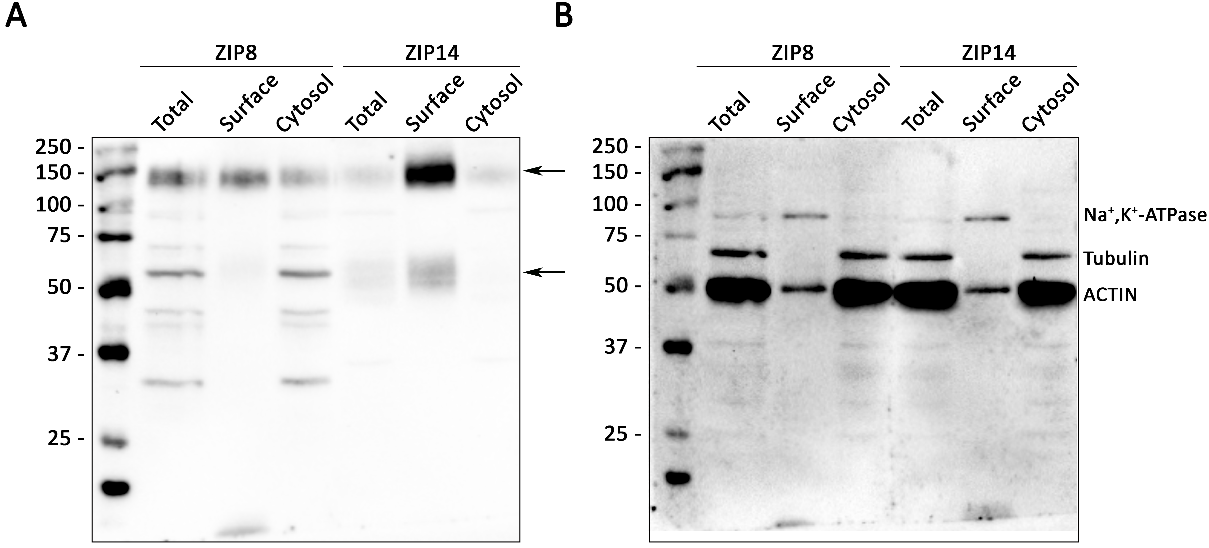
**

**Figure S8.** Uncropped immunoblots for Figure 6B. Whole blots for (**A**) ZIP8, (**B**) Na^+^, K^+^-ATPase and Tubulin. Specific bands for ZIP8 and ZIP14 (monomers and multimers) are indicated by arrows.


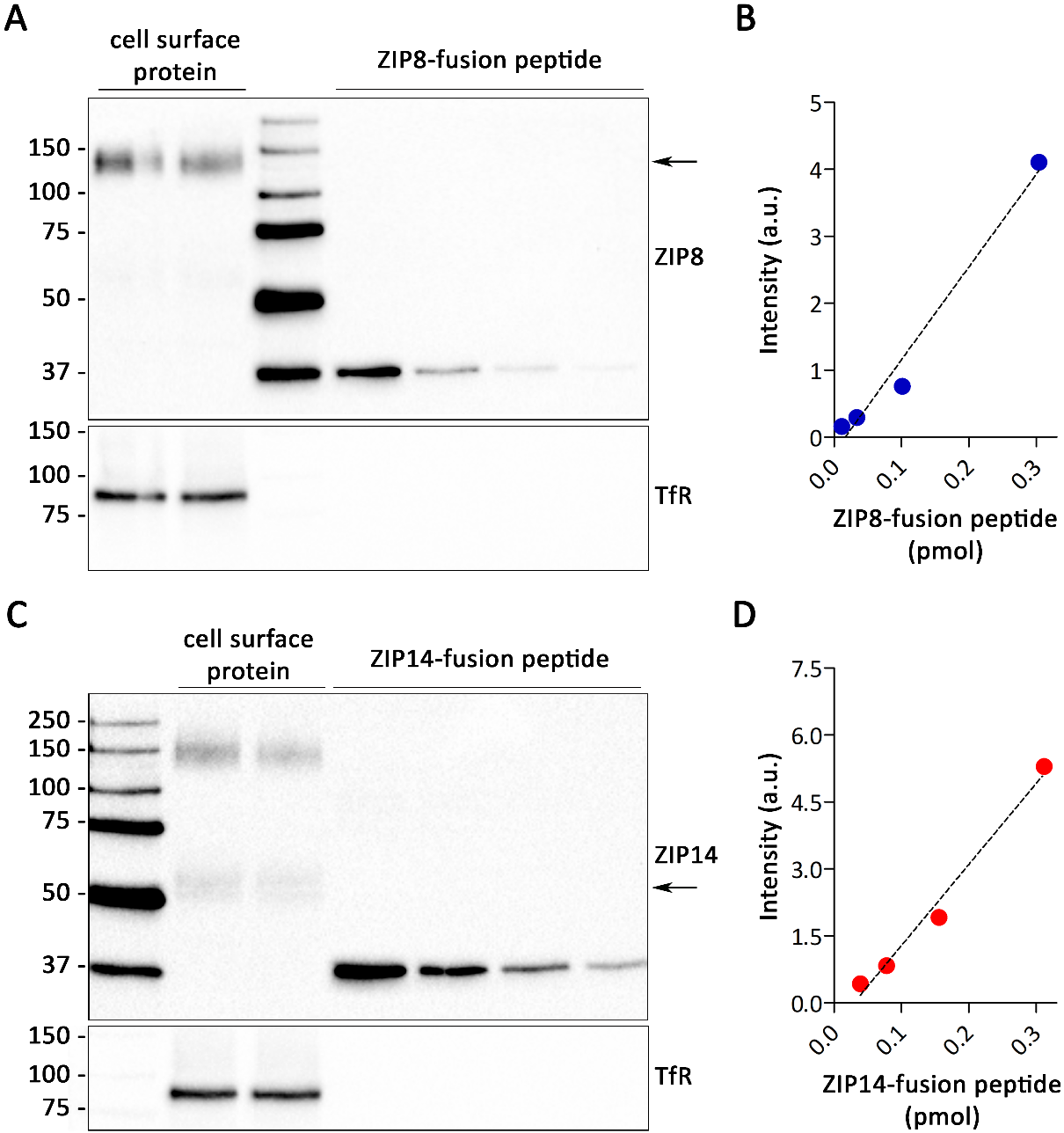


**Figure S9.** Quantification of ZIP8 and ZIP14 at the cell-surface of A549 cells. A549 cultures were subjected to surface biotinylation with cell membrane impermeable Sulfo-NHS-SS-biotin and biotin-labeled cell-surface proteins were isolated from whole-cell lysates using NeutrAvidin agarose beads. 30 μg of biotin-labeled cell-surface proteins were used for quantification of ZIP8 and ZIP14. (**A**) ZIP8 immunoblot of biotin-labeled cell-surface proteins and ZIP8-fusion protein. Specific bands for ZIP8 are indicated by arrows. Tranferrin receptor 1 (TfR) served as loading control. Band intensities for ZIP8 were quantified by densitometric analysis. (**B**) Calibration curve for ZIP8 using the ZIP8-fusion protein as a standard. (**C**) ZIP14 biotin-labeled cell-surface proteins and ZIP14-fusion protein. Specific bands for ZIP14 (monomers and multimers) are indicated by arrows. Tranferrin receptor 1 (TfR) served as loading control.Band intensities for ZIP14 were quantified by densitometric analysis. (**D**) Calibration curve for ZIP14 using the ZIP14-fusion protein as a standard. Data are from representative experiments.
